# Use of a radioactive check device for redundancy check of ionization chambers

**DOI:** 10.1120/jacmp.v1i4.2636

**Published:** 2000-09-01

**Authors:** N. P. S. Sidhu, Alkis Rouvas, Patrick Cadman

**Affiliations:** ^1^ Department of Medical Physics, Saskatoon Cancer Centre, 20 Campus Drive University of Saskatchewan Saskatoon Saskatchewan S7N 4H4 Canada

**Keywords:** constancy check, redundancy check, radioactive check device, strontium 90 check device

## Abstract

The use of a radioactive check device containing Strontium 90 was investigated to carry out the redundancy checks of Farmer‐type ionization chambers. It was not possible to meet the recommended tolerance limits of the redundancy checks without taking into account the angular response of the ionization chambers. The ionization chambers exhibited a maximum variation of 1% in the angular response in this check device. After accounting for the angular response the maximum variation of the short‐term repeatability was 0.14% with a standard deviation of ±0.05%. The long‐term constancy checked over a period of nine months was less than ±0.6% for measurements, taking into account the angular response of the ionization chambers. No significant effect of the background radiation levels was observed on the measurements.

PACS number(s): 87.53.–j, 87.52.–g

## INTRODUCTION

Redundancy in dose calibration equipment is recommended to assure that the field instruments maintain the same dosimetric characteristics as when they were calibrated.^1^ ^,^ ^2^ The redundancy checks of field ionization chambers can be carried out using either a radioactive check device with a fixed geometry or by maintaining two independent dosimetry systems as shown by Rozenfield and Jette.^3^ The radioactive check devices usually contain long‐lived radioisotopes such as Strontium 90 or Cesium 137, as described by de Souza *et al*.^4^ As per the AAPM Task Group 40 (TG 40), a Cobalt 60 teletherapy treatment machine cannot substitute for a fixed‐geometry radioactive check device unless it is not used to treat patients.^1^ In our clinic, we retired the Cobalt 60 treatment unit last year. This necessitated the use of alternative means to carry out the redundancy checks of our ion chambers. We investigated the use of a fixed‐geometry radioactive check device containing Strontium 90 to carry out redundancy checks of our ionization chambers. In our investigations, we studied the short‐term repeatability and the long‐term reproducibility of redundancy checks of cylindrical ionization chambers using this check device. We also investigated the amount of background radiation levels and their effect on the measurements.

## MATERIALS AND METHODS

**Figure 1:**
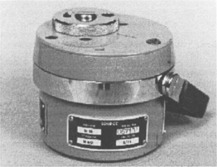
Photograph of a Sr‐90 check device.

The fixed‐geometry check device used in this study was manufactured by the Nuclear Enterprises Ltd., Reading, England. A photographic picture of the device is shown in Fig. 1. The device contains a 10 mCi Strontium 90 source in a shielded pig. The exposure rate at the surface of the device is less than 7.5 μSv/hr. The device has an approximate 7‐cm‐deep channel to accommodate a Farmer‐type ion chamber. The diameter of the channel is fixed and is sufficient to accommodate snugly an ionization chamber with a maximum outer diameter of 8.6 mm. When the device is not in use, the channel is covered by a self‐shutting, spring‐loaded, protective lid. There is a second channel in the check device to accommodate a thermometer to measure temperature close to the point of measurement. The device weighs less than 10 kg.

The ion chambers used in these investigations were two fully guarded PR‐06G and four unguarded PR‐06C ion chambers manufactured by Capintec Inc., Pittsburgh, PA. The PR‐06G ion chambers were connected to a PTW Unidos Universal Dosemeter (PTW‐Freiburg, Lorrancher Strasse 7, Germany). The PR‐06C ion chambers were each connected to a Capintec Model 192 Exposure meter (Capintec Inc., Pittsburgh, PA). The PTW Unidos measured the ionization charge collected in coulombs (C) and the Capintec Exposure meters provided readings in arbitrary units of “mR.” Both types of ion chambers have similar structural design. The outer diameter of these ion chambers at the distal end was 7 mm, to a length of 2.4 cm, gradually increasing to a maximum of 8.6 mm. Due to this gradual increase in the diameter, these ion chambers fit quite snugly and reproducibly in the channel of the check device. It took about 45 seconds to collect a charge of 1 nC, in this check device.

## RESULTS AND DISCUSSION

**Figure 2:**
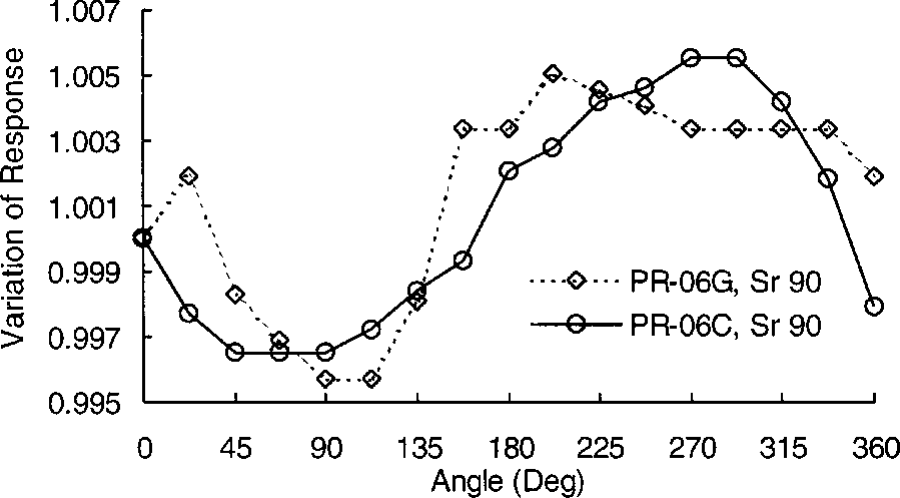
Angular response of ion chambers in the Sr‐90 check device.

The short‐term repeatability measurements were performed as described by the IEC.^5^ An ion chamber was completely removed and inserted in the check device ten times. After several failed attempts to meet the tolerance limit of ±0.3% for short‐term repeatability of redundancy check measurements as recommended by the IEC,^5^ it was decided to investigate the angular response of these cylindrical ion chambers in this check device. The angular response measurements were taken by rotating the ion chambers in the check device with respect to a fixed point marked on the check device. The axis of rotation was along the long axis of the ion chambers. The chamber responses were corrected for temperature and pressure at the time of measurement. The characteristic angular response curves of both the guarded ion chambers (PR‐06G) were the same, and the shapes of the angular response curves for all the unguarded (PR‐06C) ion chambers were similar. Typical response curves for a PR‐06G and a PR‐06C ion chamber as measured in this check device are shown in Fig. 2.

**Figure 3:**
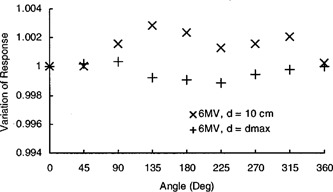
Angular response of the ion chamber in a 6 MV beam, $10\times 10\;{\text{cm}}^{2}$, SSD=100 cm.

As shown in this figure, there are some differences between the characteristic angular response curves of the guarded and unguarded ion chambers. The difference between the characteristic shapes of the angular response curves of these two types of ion chambers with otherwise similar structural design is due to the somewhat asymmetric shape of the guard electrode, as pointed out by the manufacturer. As shown here, the ion chambers demonstrated a maximum variation of about 1% in angular response in the check device using a Strontium 90 source. An anisotropy in the angular response of cylindrical chambers is usually due to an eccentricity of the central electrode or a marked nonuniformity of the wall thickness.^6^ The magnitude of the anisotropy in the angular response of cylindrical chambers, therefore, would be expected to vary with beam quality and dose gradient over the active diameter of an ion chamber. To confirm this, the angular response measurements for these ion chambers were also made in a 6 MV beam using a $10\times 10\;{\text{cm}}^{2}$ field size at depths of 10 cm and dmax in a water phantom with a source to axis distance of 100 cm. As shown in Fig. 3, the maximum variation in the angular response of these ion chambers in a 6 MV beam in the nonzero dose gradient region (at depth 10 cm) is about 0.3% and in the zero dose gradient region (at dmax) is only 0.1%. This marked variation in angular response exhibited in this check device is mainly due to the design of this check device. The pure beta‐emitter Strontium 90 radioactive source is mounted close to the channel used to insert an ion chamber in the check device. The beta particles from the source irradiate an ion chamber from one side only. This creates a very steep dose gradient over the active diameter of an ion chamber. Due to this steep dose gradient the ion chambers exhibit marked anisotropy in their angular response in this check device.

Because of this anisotropic angular response exhibited by these ion chambers in this check device the short‐term repeatability measurements were then made by aligning a point on the ion chambers with a fixed point on the check device. The maximum variation of the short‐term reproducibility measurements was 0.14% with a standard deviation σ of ±0.05% (for ten measurements). The short‐term repeatability measured this way is well within the limits of ±0.3% as recommended by the IEC.

**Figure 4:**
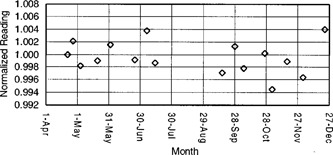
Long‐term constancy of a PR‐06G ion chamber.

The long‐term reproducibility measurements were made by two methods. In the first method, a point marked on the ion chamber was always aligned with a fixed point marked on the check device. In the second method, the measurements were made by inserting an ion chamber in a random angular orientation without aligning a point on the ion chamber with a fixed point on the check device. The measurements were then performed by successively rotating the ion chamber by 90 degrees, three times, relative to the initial random position. The measurements taken this way at four angular positions, 90 degrees apart, were then averaged. The measurements taken using the second method check an ion chamber from all axial directions and are free from procedural errors such as a loss of the mark on the ion chamber, over a long period of time. The long‐term reproducibility measurements were repeated at least twice a month for eight months over a total time span of nine months. We missed the measurements for a month during this period. The long‐term constancy measurements were corrected for temperature and pressure as well as for the decay of the Strontium 90 radioisotope. The results of long‐term reproducibility for a fully guarded (PR‐06G) ion chamber are shown in Fig. 4. These results are the average of the measurements taken at four successive rotation angles (90 degrees apart) from an initial random angular position of the ion chamber in the check device.

The maximum variation of the long‐term reproducibility is less than ±0.6% and is within the limits of ±0.7%, as recommended for such ion chambers and a check device.^5^ ^,^ ^6^ The results for long‐term constancy as measured by aligning a point on the ion chamber with a fixed point on the check device did not show any significant difference from these measurements. The reason for no significant difference between two types of measurements for long‐term constancy may be attributed to an almost sinusoidal shape of the angular response curve exhibited by these ion chambers.

In order to check the effect of scattered radiation from nearby objects surrounding the check device, the measurements were made by surrounding the check device using two 4‐cm‐thick lead bricks. The measurements that were made with and without the lead bricks surrounding the check device did not show any significant difference. This results from Strontium 90 being a pure beta emitter producing very small leakage effects. The exposure rate at the surface of the device is less than 7.5 μSv/hr. As the radiation source in this check device is placed very close to the measuring channel, only backscattered radiations scattering through 180 degrees will be able to enter the sensitive volume of an ionization chamber in the device. Considering about 80% attenuation of the backscattered radiations in the shielding walls of this device, the background radiation levels entering the measuring channel will be at 1.3 mR/min. These background radiation levels will produce a signal of about 0.24 pC in a Farmer‐type ionization chamber with a sensitive volume of 0.6 cc. This background signal is much smaller than a typical signal of 1.33 nC/min observed for these ionization chambers in this check device.

## CONCLUSIONS

We found the use of a radioactive check device for redundancy checks of our ion chambers quite user friendly. Due to its easy transportability and no significant effect of surrounding objects on the measurements, it is possible to carry out the redundancy checks of the ion chambers in treatment rooms where some of our ion chambers are permanently located for daily output checks. It is, however, important to take into account the effect of the angular response exhibited by the cylindrical ion chambers in this check device in order to meet the recommended limits for these tests. The recommended limits for the short‐term repeatability and long‐term reproducibility can be met by always inserting an ion chamber in the check device with the same orientation. The limits for the long‐term reproducibility can also be met by taking measurements at four rotation angles, 90 degrees apart, from an initial random position, and averaging the readings. The latter method has the advantage of checking an ion chamber from all axial directions.

## ACKNOWLEDGMENTS

The authors would like to thank Dr. Douglas Cormack and Dr. William Ziegler for many useful discussions.
